# Periodic sea-level oscillation in Tokyo Bay detected with the Tokyo-Bay seafloor hyper-kilometric submarine deep detector (TS-HKMSDD)

**DOI:** 10.1038/s41598-022-10078-2

**Published:** 2022-04-12

**Authors:** Hiroyuki K. M. Tanaka, Masaatsu Aichi, Szabolcs József Balogh, Cristiano Bozza, Rosa Coniglione, Jon Gluyas, Naoto Hayashi, Marko Holma, Jari Joutsenvaara, Osamu Kamoshida, Yasuhiro Kato, Tadahiro Kin, Pasi Kuusiniemi, Giovanni Leone, Domenico Lo Presti, Jun Matsushima, Hideaki Miyamoto, Hirohisa Mori, Yukihiro Nomura, Naoya Okamoto, László Oláh, Sara Steigerwald, Kenji Shimazoe, Kenji Sumiya, Hiroyuki Takahashi, Lee F. Thompson, Tomochika Tokunaga, Yusuke Yokota, Sean Paling, Dezső Varga

**Affiliations:** 1 University of Tokyo, Tokyo, Japan; 2 The University of Salerno, Salerno, Italy; 3 Istituto Nazionale Di Fisica Nucleare-Laboratori Nazionali del Sud, Catania, Italy; 4 Durham University, Durham, UK; 5 Kerttu Saalasti Institute, University of Oulu, Oulu, Finland; 6 Muon Solutions Oy Ltd, Saarenkylä, Finland; 7 Arctic Planetary Science Institute, Rovaniemi, Finland; 8 NEC Corporation, Tokyo, Japan; 9 Kyushu University, Fukuoka, Japan; 10 The University of Atacama, Copiapó, Chile; 11 The University of Catania, Catania, Italy; 12 Istituto Nazionale Di Fisica Nucleare, Catania, Italy; 13 The University of Sheffield, Sheffield, UK; 14 Geoptic Ltd, Warnborough, UK; 15 Boulby Underground Laboratory, Saltburn-by-the-Sea, UK; 16 Wigner Research Centre for Physics, Budapest, Hungary; 17 International Virtual Muography Institute (VMI), Global, Tokyo, Japan; 18 Chiba University, Chiba, Japan

**Keywords:** Ocean sciences, Experimental particle physics

## Abstract

Meteorological-tsunami-like (or meteotsunami-like) periodic oscillation was muographically detected with the Tokyo-Bay Seafloor Hyper-Kilometric Submarine Deep Detector (TS-HKMSDD) deployed in the underwater highway called the Trans-Tokyo Bay Expressway or Tokyo Bay Aqua-Line (TBAL). It was detected right after the arrival of the 2021 Typhoon-16 that passed through the region 400 km south of the bay. The measured oscillation period and decay time were respectively 3 h and 10 h. These measurements were found to be consistent with previous tide gauge measurements. Meteotsunamis are known to take place in bays and lakes, and the temporal and spatial characteristics of meteotsunamis are similar to seismic tsunamis. However, their generation and propagation mechanisms are not well understood. The current result indicates that a combination of muography and trans-bay or trans-lake underwater tunnels will offer an additional tool to measure meteotsunamis at locations where tide gauges are unavailable.

## Introduction

Meteotsunamis or meteorological tsunamis are tsunami-like sea-level oscillations that take place in closed or semi-closed water bodies in bays or lakes with periods ranging from minutes to several hours^1^, As the generation of these tsunamis is related to one of the natural oscillation modes of the bay or the lake, their period and amplitude are a function of the size, depth and the configuration of the coastline^2^. The temporal and spatial characteristics of meteotsunamis and seismic tsunamis are similar, and shifting atmospheric disturbances, which are usually caused by sudden atmospheric pressure and/or wind changes, are the significant factors that will induce oscillations in water bodies. Atmospheric energy is transferred to a body of water more efficiently and in a more concentrated manner when the propagation speed of the atmospheric disturbance is approximately equal to the local free wave speed. This process is even more efficient if the water depth is within the optimal range^3^. Stronger atmospheric disturbances usually generate larger scale oscillations. Meteotsunamis are associated with frontal passages^4^, cyclones^5,6^, atmospheric gravity waves^7^, and mesoscale convective systems^8,9^, including derechos^10^, and have been reported worldwide^1,11^. Importantly, their impacts on human communities and infrastructure are often severe ^11^ due to their high wave runup and strong associated currents^12–15^. Even meteotsunamis with moderate heights (~ 0.3 m) generate hazardous currents^16,17^. Owing to the ubiquitous nature of atmospheric disturbances, associated meteotsunamis can add to the risk posed by seismic tsunamis^18^ or can increase the risk to regions not traditionally recognized as seismic-tsunami-prone^19^. However, our quantitative understanding of the associated risks and frequency, generation, and propagation mechanisms related to meteotsunamis is limited^20,21^.

A typhoon is defined as a tropical cyclone (TC) when it develops in the Northwestern Pacific Basin, which has been recognized as one of the most active tropical cyclone areas in the world^22^. In Japan, typhoon measurements have been recorded since 1951^23^. In Tokyo Bay, meteotsunamis induced by typhoons and the sea surface currents induced by meteotsunamis have been respectively measured with a tide gauge^24^ and High Frequency Radar (HFR)^25^. Results from these measurements are consistent with each other, and have differentiated two oscillation modes (OM) in Tokyo Bay, more specifically, OM1 (usually 2–3 h duration) and OM2 (usually 5–6 h duration). It has been interpreted that OM1 occurs when the tsunami is confined in the northern part of Tokyo Bay, whereas OM2 is associated with the tsunamis that occur throughout the entire longitudinal length of Tokyo Bay^25^.

Muography is similar to x-ray imagery, but it utilizes the strong penetration capability of high-energy muons (> a few tens of GeV) and their relativistic effect. Since the number of muons that pass through gigantic bodies reflects the interior spatial distribution of density, this distribution can be mapped by identifying where these muons passed through the object and subsequently creating a plot of the number of penetrating muons on a 2-dimensional plane. The origin of these high-energy muons is galactic cosmic rays (GCR) which are accelerated by high-energy events such as supernovas in our galaxy. The GCRs mainly consist of protons and alpha particles. These charged particles are generally accelerated to close to the speed of light. Although the galactic magnetic field is mainly aligned with the spiral galaxy, there is also a random component. The direction that cosmic rays travel is strongly affected by this random component of the galactic magnetic field. As a result, cosmic rays travel for millions of years (depending on their energy) before arriving at the Solar System. Consequently, by the time they arrive here, their initial direction of origin is completely lost as they have obtained an isotropic distribution of arrival directions. These cosmic rays interact with the Earth's atmosphere, and muons are generated. Muography takes advantage of the characteristics of muons, particularly their penetrative nature and universality, for a wide variety of applications, including visualizing the internal structure of volcanoes, tunnels, natural caves, and cultural heritage. So far, applications have focused on targets in Africa^26,27^, the Americas^28–30^, Asia^31–41^, and Europe^42–48^.

The Tokyo-Bay Seafloor Hyper-Kilometric Submarine Deep Detector (TS-HKMSDD) consists of a linear array of several particle detectors located inside the underwater tunnel called the Tokyo Bay Aqua-Line (TBAL). The first and second segments of TS-HKMSDD were respectively installed in March 2021^23^ and June 2021. The current total length of TS-HKMSDD is 200 m with a total active area of 3 m^2^. This article reports the results found thus far, including a meteotsunami-like periodical oscillation in muon flux as observed with TS-HKMSDD right after a typhoon approached Tokyo.

## Results

**Figure 1:**
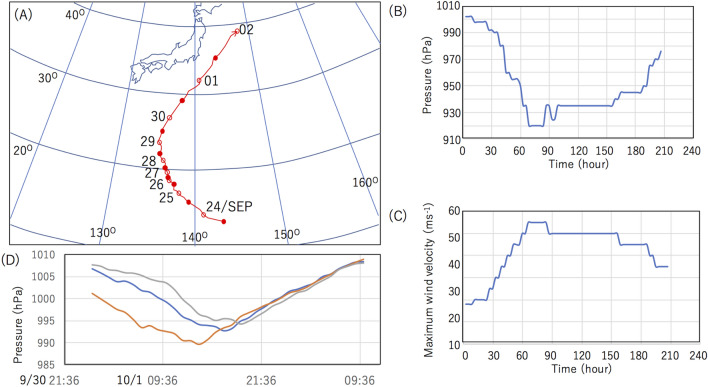
Meteorological history of 2021-Typhoon-16. The trajectory of the Typhoon-16 (**A**), the central pressure (**B**), and the maximum wind velocity (**C**) are shown as a function of the time since the moment of its development on September 24. The severe pressure drops observed on October 1 in Irozaki (orange), Tokyo Bay (Yokohama, blue), and Mito (gray) are also shown (**D**). HKMT drew the map based on the data in Reference^23^ and the image with Microsoft PowerPoint software and holds the copyright.

The 2021-Typhoon-16 originated at 13.6° N and 143.3° E at 21:00 September 23, 2021. Figure 1A–C show the meteorological history of Typhoon-16. The Japan Meteorological Agency (JMA) reported that this typhoon tracked first westward but transiently shifted to a more northerly course on September 26^23^. At 09:00 on September 26, Typhoon-16 was estimated to have attained Category 3 winds (178–208 km/h). At 15:00 JST on September 26, Typhoon-16 achieved its minimum barometric pressure at 920 hPa. This fall in barometric pressure indicated a 78 hPa pressure drop in the preceding 48 h. The typhoon's winds continued to increase before peaking at 15:00 JST on September 26, with its maximum wind velocity reaching 198 km/h^23^. Typhoon-16 only maintained peak intensity for 15 h, but even after this, it remained a powerful tropical cyclone^23^. With very little change in barometric pressure from 920 to 945 hPa, the typhoon was tracked to be moving in a northeastward direction throughout October 1. Meanwhile, the storm area (> 54 km/h) had grown from E: 390 km W: 280 km to E: 750 km W: 560 km. At that moment, Typhoon-16 was located 400 km south of Tokyo. Following this approach of Typhoon-16 to Tokyo Bay, the barometric pressure observed in Yokohama, Japan, had dropped by ~ 20 hPa in 24 h between 17:00 JST on September 30 and 17:00 JST. Figure 1D shows the sequence of the pressure drops observed in the cities (Irozaki, Yokohama and Mito) that are sparsely located on a SW-NE line on October 1. Since the linear distance between Irozaki and Yokohama and that between Yokohama and Mito are both 120 km the average speed between Irozaki and Yokohama and that between Yokohama and Mito were 40 kmh^-1^ and 60 kmh^-1^, respectively. Considering the depth of Tokyo Bay ranges 15–20 m, the free wave speed would be 44–50 kmh^-1^ in Tokyo bay. At 21:00 JST on October 1, the typhoon had weakened to tropical storm intensity and transformed into an extratropical cyclone at 09:00 JST on October 2.

**Figure 2:**
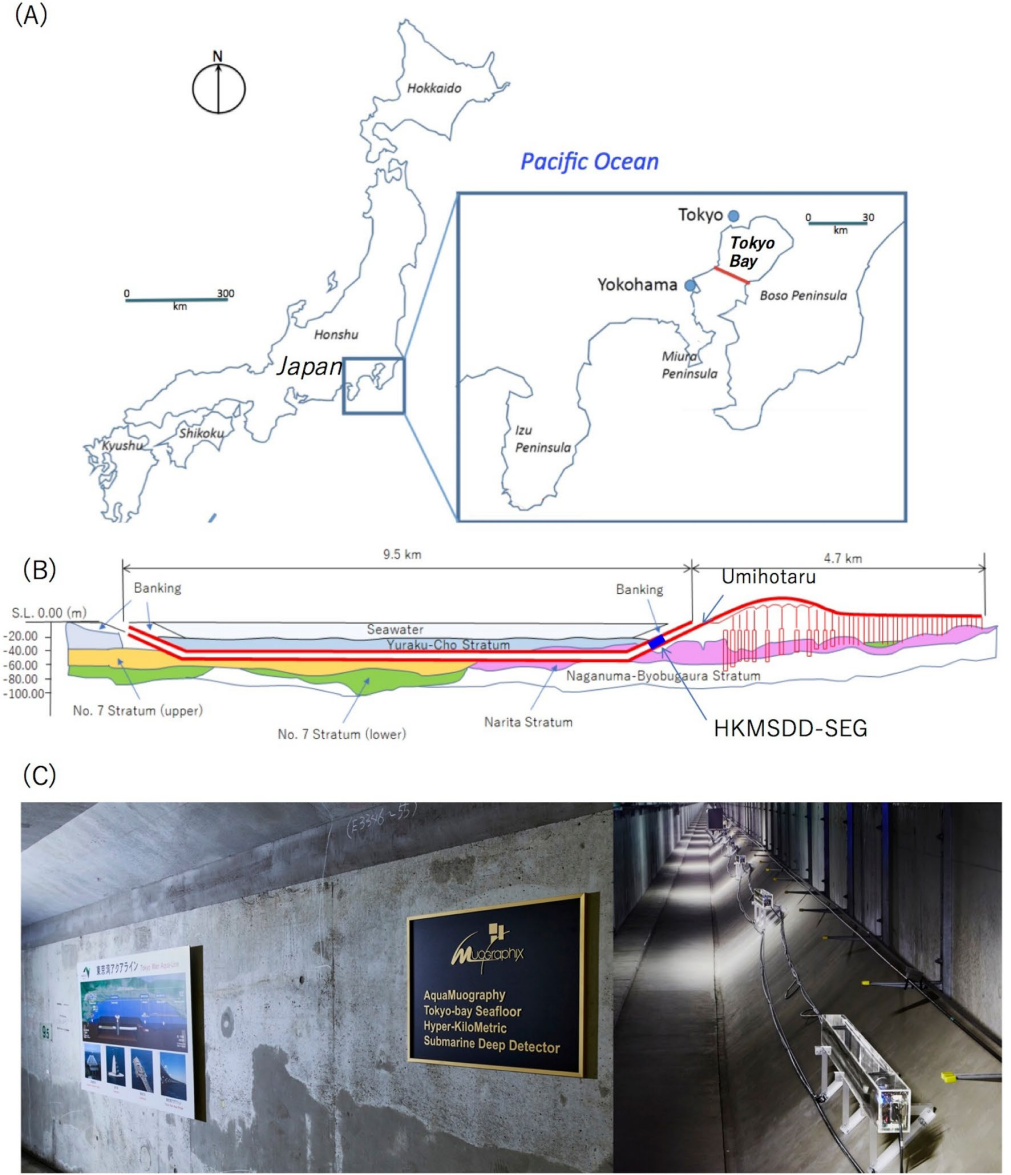
Location of the Tokyo Bay Aqua-Line (TBAL). The inset shows the magnified view of the southern central part of Japan that includes Tokyo Bay (**A**). The red line indicates the location of TBAL. The cross-sectional view of TBAL is shown in the middle panel (**B**). The symbol "HKMSDD-SEG" indicates the location of the currently installed TS-HKMSDD that spans 200 m along the tunnel. The photograph of TS-HKMSDD is also shown (**C**). HKMT drew the map and the image with Microsoft PowerPoint software and holds the copyright of the images and photographs.

The Trans-Tokyo Bay Expressway, also known as TBAL, is a combined bridge and tunnel structure spanning the entire width of Tokyo Bay, Japan (Fig. 2). It consists of a 4.4-km long bridge and a 9.6-km long tunnel underneath the bay. The tunnel section is called the Aqua-Tunnel. The average sea depth is 20 m in most of the region where the Aqua-Tunnel was constructed. The tunnel was constructed at a depth of 20 m underneath the seafloor. In this work, 20 muographic sensor modules (MSMs) were deployed inside the Aqua-Tunnel to construct a linear array of MSMs called the Tokyo-Bay Seafloor Hyper-Kilometric Submarine Deep Detector (TS-HKMSDD). Since a detailed description of HKMSDD can be found elsewhere^49^, it is only briefly introduced here. Each MSM consisted of two scintillation detectors, a high-voltage power supply unit (HVU) (Technoland Z-SYS 070HV), and a discriminator-coincidence unit (DCU) (Technoland Z-SYS 070DC), and only the events producing signals at both detectors in coincidence were considered muon events. Each scintillation detector consists of a plastic scintillator (ELJEN EJ-200) that measures 20 mm in thickness, 100 mm in width and 1500 mm in length that is coupled with a 2-inch photomultiplier tube (PMT) (HAMAMATSU H7195) via an acrylic light guide. An HKMSDD segment consists of 10 MSMs with an interval of 10 m and the data acquisition center (DAC) located at the center of the segment. The DAC was installed to the 19-inch rack for mounting the data acquisition (DAQ) electronics, and this 19-inch rack was placed in a box to protect it from the dust in the local environment. All of the MSMs are anchored to the tunnel wall with bolts and frames to fix their position, and each MSM is connected to DAC with the water-resist D-SUB cables (IP67). Two HKMSDD segments were used in the current work. The discriminated and logically processed signals output from MSMs are processed by four complex programmable logic devices (CPLD) (Intel 10M08) and a microcomputer board (Raspberry Pi 4) is used for sending the time-sequential muon count data to the external server via an 8-core optical fiber cable. The network speed was 1 Gbps on a best-effort basis. A 2-cm-thick lead block was inserted between these plastic scintillators to reduce the number of random coincidences resulting from electronic noise or the gamma-rays emitted from the tunnel's concrete wall. The temperature values are monitored in the vicinity of the detectors respectively located at the locations closest and furthest from the tunnel entrance as well as inside the DAC. Although the temperature measured in the DAC was slightly higher than that outside DAC, the daily temperature variations were suppressed less than 2 °C in the current underwater tunnel.

**Figure 3:**
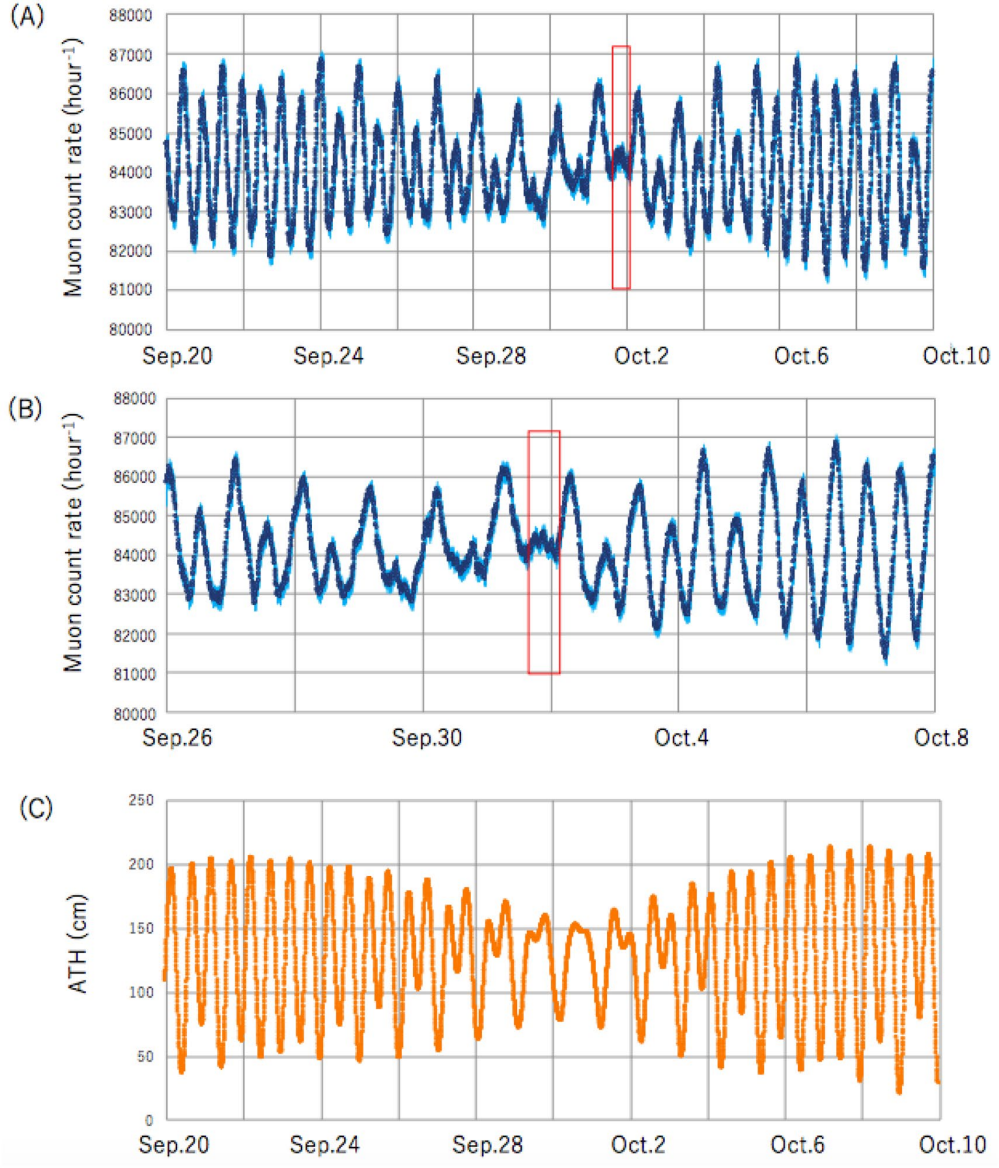
Muon count rate (MCR) as recorded with TS-HKMSDD. The time series plots are shown for 3 weeks (**A**) and 12 days (**B**), including the period when Typhoon-16 approached and occupied the region 400 km south of Tokyo Bay. The astronomical tide height (ATH) variations (**C**) are also shown for the same period as in Panel A. The red boxes indicate the time region when the periodic oscillation was observed right after the pressure drop observed in Tokyo Bay.

Figure 3A shows the muon count rate (MCR) as recorded every 5 min at TS-HKMSDD for 3 weeks, including the period when Typhoon-16 approached and occupied the region 400 km south of Tokyo Bay. At TS-HKMSDD, a large portion of the inverse barometric effect (IBE) is cancelled except for the small residual IBE coming from the muon's different energy loss rate between air and water^53^. There had been a clear anti-correlation with the astronomical tide height (ATH) variations (Fig. 3C) except for the period right after the severe atmospheric pressure drop observed at Tokyo Bay on October 1. Figure 3B focuses on the MCR data recorded for 12 days including the period when Typhoon-16 approached the region 400 km south of Tokyo Bay. As can be seen in the red box of Fig. 3B, this disturbance was an oscillation with a period of ~ 3 h. The starting point and the duration of the red box were respectively corresponding to the timing when the minimum pressure was observed in Yokohama (17:00 JST on October 1) and the oscillation decay time (10 h) calculated based on the damping coefficient (2.45 × 10^–5^ s^-1^) of Tokyo Bay that was modelled for the oscillation observed during the passage of Typhoon-15 on September 11, 2001^24^.

## Discussion

**Figure 4:**
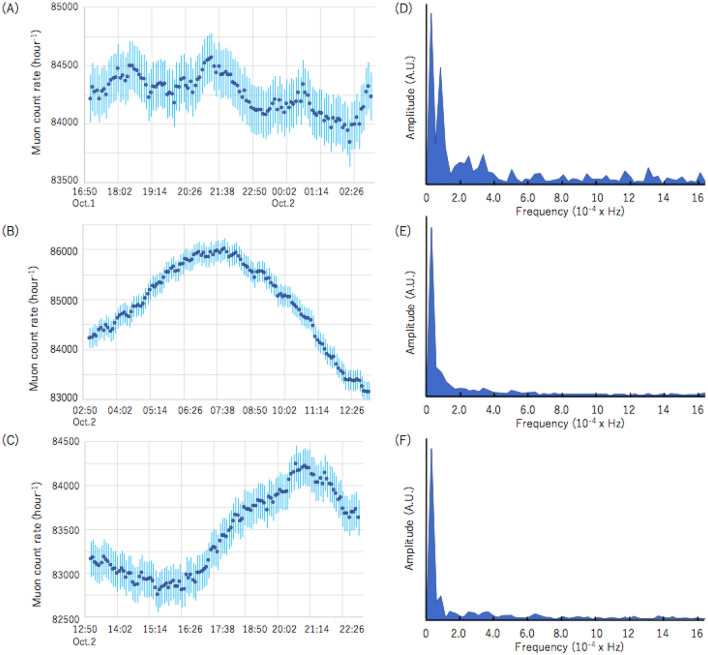
Time series showing the muon count rate (MCR) in different time windows (**A–C**) and their Fast Fourier Transformation (FFT) results (**D–F**). Vertical bars in (**A–C**) indicate the one standard deviation error bars.

*Oscillation decay time* Figure 4A focuses on the time region within the red box in Fig. 3. The observed oscillation period was ~ 3 h and is consistent with the period (155 min) measured right after the passage of 2015-Typhoon-15^25^, which was associated with the confined mode (OM1). Figure 4B, C focuses on two other periods that follow the time region shown in Fig. 4A (03:00–13:00 on October 2 and 13:00–23:00 on October 2).

**Figure 5:**
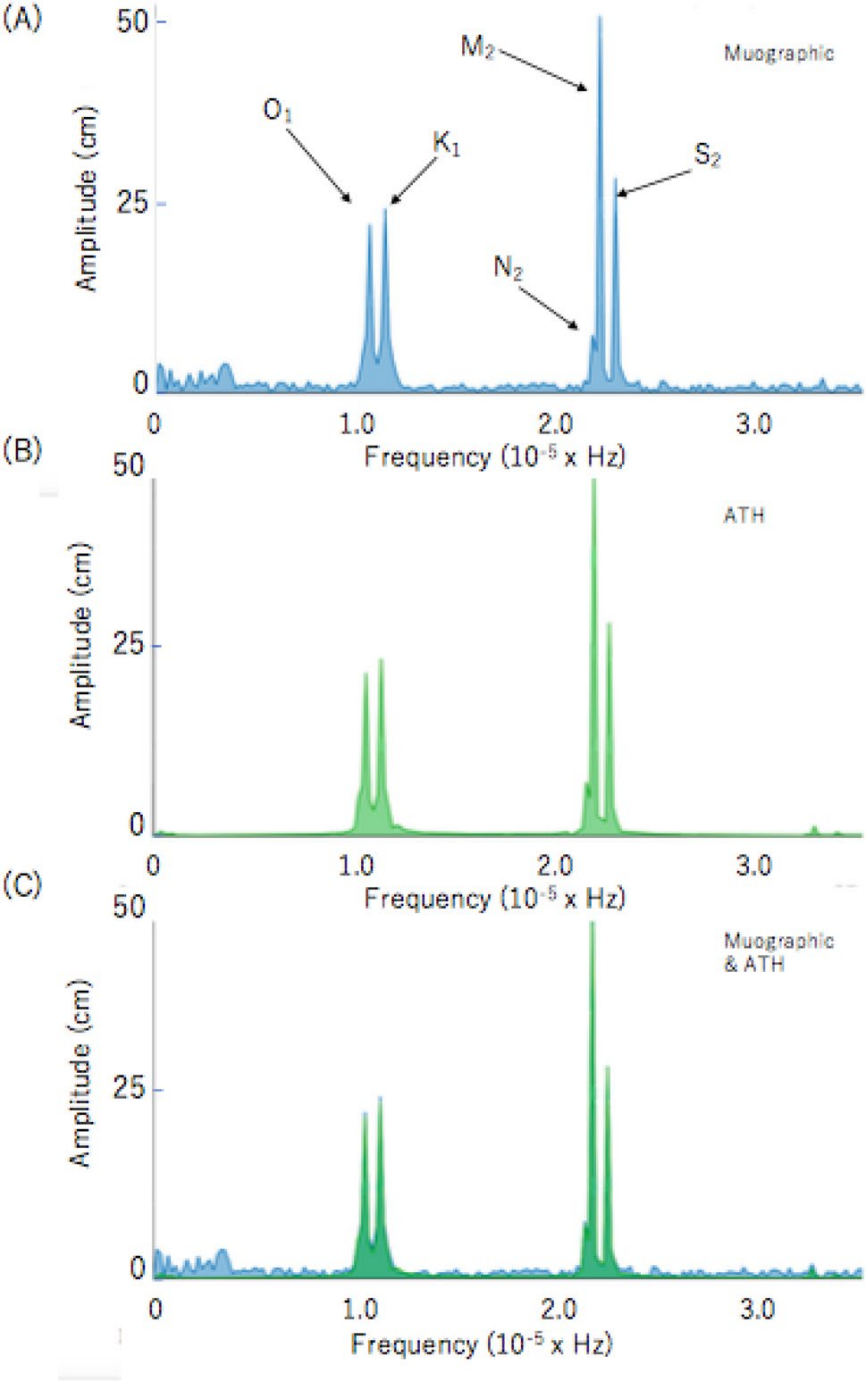
Power spectra of MCR (**A**) and ATH (**B**), recorded between August 1 and September 30. The power spectrum of MCR is superimposed to that of ATH (**C**). Symbols O_1_, K_1_, N_2_, M_2_, and S_2_ respectively indicate the lunar diurnal A, lunar diurnal B, larger lunar elliptic semi diurnal, principal lunar semi diurnal, and principal solar semi diurnal constituents. In these plots, the amplitude of the muographic M_2_ peak in (**A**) was normalized to the astronomical M_2_ peak (**B**).

Figure 4D–F show the result of the Fast Fourier Transformation (FFT) of MCR recorded in the time regions shown in Fig. 4A–C, respectively. An oscillation with a frequency of ~ 100 micro-Hz (a period of ~ 3 h) can be seen only in Fig. 4D. The first peak seen in all of the figures (Fig. 4D–F) is associated with ATH (for more details, see Fig. 5).

For validation of the current FFT computation, Fig. 5 compares the results of the FFT of MCR and ATH^49^ as they were recorded during the previous 2 months (August 1–September 30). Four fundamental tidal constants are well reproduced, and the spectrum shapes are almost identical between MCR and ATH. The tidal constituents were those of lunar diurnal A (O_1_), lunar diurnal B (K_1_), principal lunar semidiurnal (M_2_), and principal solar semidiurnal (S_2_). Figure 5 essentially shows five major tidal constituents of tide levels at Tokyo Bay, Japan^50^.

**Figure 6:**
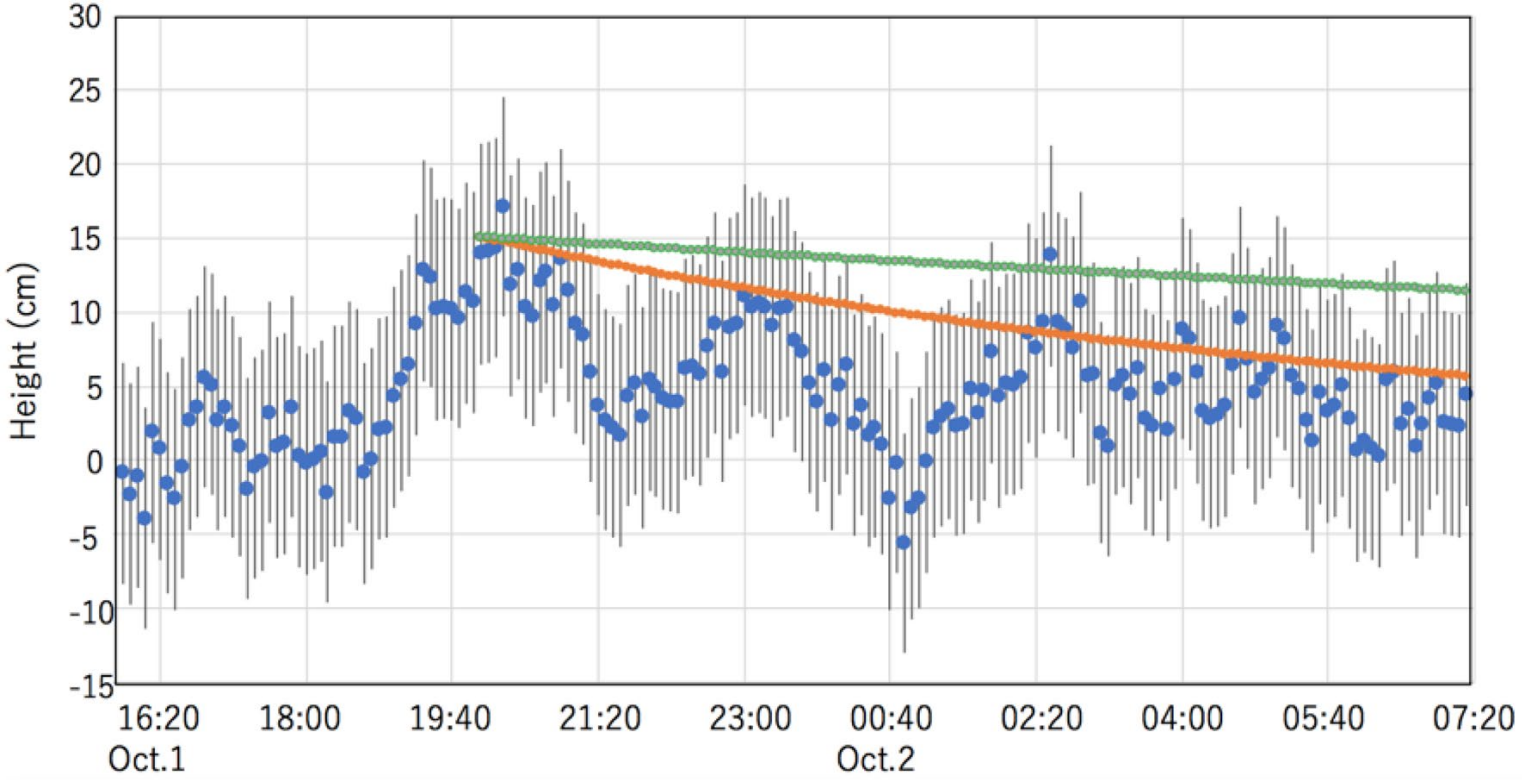
Time series of the abnormal tides muographically measured between 15:50 on October 1 and 07:20 on October 2, 2021. The decay curves calculated for Tokyo Bay (orange) and Lake Geneva (green) are overlaid. Vertical bars indicate the standard deviation error bars.

Figure 6 shows the time series of the abnormal tides measured muographically between 15:50 on October 1 and 07:20 on October 2, 2021. These time series were derived by subtracting ATH from the tide levels converted from MCR^49^. The decay curves (Eq. 1) with a damping coefficient modeled for Tokyo Bay and Lake Geneva are overlaid.

1 $$H = H_{0} {\text{exp}}( - \beta t),$$

where *H* is the time-dependent tide height, *H*_0_ is 15 m, the damping coefficient *β* is 2.43 × 10^–5^ s^-1^ for Tokyo Bay and 6.83 × 10^–6^ s^-1^ for Lake Geneva^25^. Based on Figs. 4, 5 and 6, it can be concluded that the oscillation decay time is consistent with the value previously modeled for Tokyo Bay. The decay time of the oscillation observed here was consistent with the Tokyo-bay's damping coefficient estimated in the prior work^24^. Also, the currently observed decay time was much shorter than what was observed in Lake Geneva. Lake Geneva's oscillation period and depth were respectively 70 min and 150 m while Tokyo Bay's oscillation period was respectively 3 h and 15 m; this shorter decay time matches the meteotsunami damping model proposed by Kinari^24^:

$$\beta^{{2}} \sim 0.{25}\left( {hT} \right)^{{ - {1}.{2}}} ,$$

where *h* (m) and *T* (minutes) are respectively depth and period.

In conclusion, it has been shown that muography conducted inside an underwater tunnel has the potential to become a valuable tool for monitoring meteotsunamis in bays and lakes in regions where no other measurement tools are available. Understanding the regional tides of inner bays is not only vital for navigation safety, but also necessary for environmental hazard assessments. The concept of HKMSDD is deployable at any given underwater tunnel of the appropriate size and depth worldwide. For example, the Transbay Tube at San Francisco Bay, CA, where a well-known meteotsunami event was induced by a moving pressure pulse on November 21, 1910^51^, has similar characteristics to the TBAL site currently used for TS-HKMSDD. Other meteotsunami examples can be found in the English Channel^9,52^ and the Gulf of Finland^53^. The Channel Tunnel connects the UK to France and could be used for similar measurements. Similarly, the underwater tunnel across the Gulf of Finland to connect Finland and Estonia could be a good candidate location if the tunnel project is realized. Various trans-bay and under-lake tunnels exist or are under construction globally. It is anticipated that the same muography HKMSDD configuration could be installed in several underwater tunnels worldwide to serve as a local and global sea level monitoring array.

## Supplementary Information

Supplementary Information.
