# Supplementary material for: Periodic sea-level oscillation in Tokyo Bay detected with the Tokyo-Bay seafloor hyper-kilometric submarine deep detector (TS-HKMSDD)
Source: Sci Rep. 2022 Apr 12;12:6097. doi: 10.1038/s41598-022-10078-2 (PMC9005544; doi:10.1038/s41598-022-10078-2)
Supplement: Supplementary file 1 — Supplementary Information. [file 41598_2022_10078_MOESM1_ESM.docx]

**MAGMA-HKMSDD Collaboration**

1. University of Tokyo, Japan

Hiroyuki K.M. Tanaka

Jun Matsushima

Yusuke Yokota

László Oláh,

Naoto Hayashi,

Hiroyuki Takahashi

Hirohisa Mori

Kenji Shimazoe

Hideaki Miyamoto

Eriko Maeda

Shouhei Hanaoka

Yukihiro Nomura

Takeharu Yoshikawa

Yoshihiro Nishiaki

Yasuhiro Kato

Masaki Satoh

Masaatsu Aichi

Takanobu Amano

2. The University of Salerno, Salerno

Cristiano Bozza

3. Istituto Nazionale di Fisica Nucleare - Laboratori Nazionali del Sud

Rosa Coniglione

Danilo L. Bonanno

4. Durham University, UK

Jon Gluyas

5. Kerttu Saalasti Institute, University of Oulu, Finland

Marko Holma

6. Muon Solutions Oy Ltd, Finland

Marko Holma

Pasi Kuusiniemi

7. Arctic Planetary Science Institute

Marko Holma

Pasi Kuusiniemi

8. NEC Corporation, Tokyo, Japan

Shin-ichi Miyamoto

Norimasa Kumagai

Kazuhiro Miyazawa

Yoshio Ishizawa

Ryuta Asami

Osamu Kamoshida

Toru Takeno

Tetsu Oosaki

9. Kyushu University, Japan

Tadahiro Kin

Naoya Okamoto

10. The University of Atacama, Chile

Giovanni Leone

11. The University of Catania, Catania, Italy

Domenico Lo Presti

Giuseppe Gallo

Franco Riggi

Paola La Rocca.
Carmelo Ferlito

12. Istituto Nazionale di Fisica Nucleare, Catania, Italy.
Domenico Lo Presti

Franco Riggi

Paola La Rocca.

Danilo L. Bonanno

13. Kansai University, Osaka, Japan

Takefumi Hayashi

Kenji Sumiya

14. The University of Sheffield, UK

Lee F. Thompson

Patrick Stowell

Samuel J Fargher

15. Geoptic Ltd., UK

Chris Steer

Jon Gluyas

Lee F. Thompson

Patrick Stowell

16. Boulby Underground Laboratory, UK

Sean Palling

Paul Scovell

Christopher Toth

17. Wigner Research Centre for Physics, Hungary

Dezső Varga,

Gábor Galgóczi,

Ádám L. Gera,

Gergő Hamar,

Szabolcs J. Balogh,

Gábor Nyitrai

18. International Virtual Muography Institute (VMI)

Hiroyuki K.M. Tanaka

Jun Matsushima

Kenji Sumiya

László Oláh,

Hirohisa Mori

Tadahiro Kin

Shin-ichi Miyamoto

Osamu Kamoshida

Takefumi Hayashi

Kenji Sumiya

Hiroshi Nakajima

Sara Steigerwald

Lee Thompson

Jon Gluyas

Dezső Varga,

Gábor Galgóczi,

Ádám L. Gera,

Gergő Hamar,

Szabolcs J. Balogh,

Gábor Nyitrai

Domenico Lo Presti

Giuseppe Gallo

Cristiano Bozza

Giovanni Leone

Marko Holma

Pasi Kuusiniemi

Andrea Gimmanco

Michael Tytgat

19. Central Research Institute of Electric Power Industry, Chiba, Japan

Kimio Miyakawa

Hiroshi Suenaga

20. JGI, Inc., Tokyo, Japan

Susumu Abe

Nobuo Kawai

Masazumi Onishi

Eiichi Asakawa

Takashi Imazumi

Shinji Kawasaki

Ikuro Mizukoshi

21. Japan Petroleum Exploration Co., Ltd., Tokyo, Japan

Ken'ichi Akama

Koji Kashihara

Takao Nibe

Takeshi Shibata

Katsumi Takai

22. Kawasaki Geological Engineering, Tokyo, Japan

Aya Kamimura

Osamu Fujimoto

Sunao Kanazawa

Yuki Kobayashi

Eiichiro Nishiyama

Jun Ando

Taro Kusagaya

Takehiro Ohara

Takeshi Ohmura

Hiroshi Ohnuma

Daisuke Shimokawa

Keiichi Suzuki

Akiko Ishii

Shingo Sugimoto

23. Istituto Nazionale di Astrofisica, Osservatorio di Catania, Catania, Italy
Giovanni Bonanno

Giuseppe Romeo

24. Dipartimento di Ingegneria e Geologia (INGEO), Università d'Annunzio, Italy

Piero D’Incecco

25. Istituto Nazionale di Geofisica e Vulcanologia, Catania, Italy.
Carmelo Ferlito

26. Tokyo Metropolitan University, Tokyo, Japan

Yoshiya Oda

27. Ghent University, Belguium

Michael Tytgat

28. UCLouvin, Belgium

Andrea Giammanco

29. Nagoya University, Nagoya, Japan

Hiroshi Ichihara
Toshiki Watanabe
Koshun Yamaoka

30. GEOSYS, Inc., Tokyo, Japan

Toshio Iizuka

Taro Koike

Tsukasa Nishiki

Katsuya Noda

Nobuyuki Shimizu

Kenichi Watanabe

31. Waseda University, Tokyo, Japan

Kazuo Kamura

Takumi Ueda

32. National Agriculture and Food Research Organization, Ibaraki, Japan

Seiichiro Kuroda

33. National Institute of Advanced Industrial Science and Technology, Ibaraki, Japan

Toshiyuki Yokota

34. Kyoto University, Kyoto, Japan

Yuzo Ohnishi

35. Japan Agency for Marine-Earth Science and Technology

Fumio Inagaki

Hiroshi Yoshida
